# Factors Affecting the Efficacy of Monopolar Radiofrequency in Facial Rejuvenation

**DOI:** 10.1111/jocd.71027

**Published:** 2026-07-06

**Authors:** Shasha Zhao, Yan Yan

**Affiliations:** ^1^ Department of Dermatology Plastic Surgery Hospital, Chinese Academy of Medical Sciences and Peking Union Medical College Beijing China

**Keywords:** age, facial rejuvenation, monopolar radiofrequency, pain

To the Editor,

Skin aging is a highly complex process influenced by multiple intrinsic and extrinsic factors, such as chronic ultraviolet exposure. These factors cause significant changes in facial appearance, including reduced elasticity, decreased collagen content, deep wrinkles, increased pigmentation, and a compromised skin barrier [1]. These characteristics often negatively impact a person's perceived attractiveness, self‐esteem, and overall quality of life. Radiofrequency (RF) technology is an emerging noninvasive treatment widely used for rejuvenating the face and neck [2]. Monopolar RF is currently considered the gold standard for the noninvasive treatment of skin laxity. However, there are no definitive conclusions regarding the factors that influence the efficacy of monopolar RF in facial rejuvenation.

Our previous work demonstrated that the monopolar RF device YOUMAGIC, a newly developed technology, exhibits high safety and efficacy in skin tightening [3]. The 212 subjects involved in this study included 12 males and 200 females, aged 30 to 60 years, with Fitzpatrick skin types II to V and mild to moderate skin laxity. They were randomly divided into the YOUMAGIC group (107 patients: 103 females and four males) or the Thermage group (105 patients: 97 females and eight males). In the YOUMAGIC group, the average age was 43.51 ± 7.77 years, with a baseline average Fitzpatrick Wrinkle Classification System (FWCS) score of 4.57 ± 0.93. The Thermage group had an average age of 42.73 ± 8.06 years and a baseline average FWCS score of 4.55 ± 1.02. All subjects received a single treatment session. A topical anesthetic cream (5% lidocaine) was applied for 30 min prior to treatment. Follow‐up visits occurred at 30, 90, and 180 days after the baseline visit. Three independent, blinded evaluators assessed efficacy by comparing pre‐ and post‐photographs using the Global Aesthetic Improvement Scale (GAIS). A detailed study design was presented in our previous publication [3]. To investigate potential factors influencing the effectiveness of monopolar RF in facial rejuvenation, we conducted an in‐depth analysis of the results.

The current analysis revealed that very much improvement and much improvement (GAIS score ≤ 2) were more common in younger participants (aged < 40) than in older participants (aged ≥ 40). This trend was observed in both the YOUMAGIC and Thermage groups. Six months after treatment, the difference became more pronounced. In contrast, the pain score during treatment did not affect therapeutic efficacy. At all follow‐up visits, the differences between the no‐to‐mild pain group (NRS score 0–3) and the moderate‐to‐severe pain group (NRS score 4–10) were not statistically significant. The relative risk and 95% confidence intervals are presented in Table 1.

**TABLE 1:** The impact of age and pain score on the efficacy of monopolar radiofrequency treatment.

Visit	Variables	RR	95% CI
90 days	Age (YOUMAGIC)	1.274	(1.035, 1.568)
	Age (Thermage)	1.538	(1.127, 2.099)
	Age (Total)	1.36	(1.135, 1.629)
	Pain score (YOUMAGIC)	1.168	(0.937, 1.457)
	Pain score (Thermage)	0.852	(0.62, 1.172)
	Pain score (Total)	0.995	(0.822, 1.205)
180 days	Age (YOUMAGIC)	1.811	(1.268, 2.587)
	Age (Thermage)	2.214	(1.483, 3.305)
	Age (Total)	1.985	(1.525, 2.583)
	Pain score (YOUMAGIC)	0.962	(0.653, 1.417)
	Pain score (Thermage)	0.855	(0.579, 1.262)
	Pain score (Total)	0.908	(0.689, 1.195)

*Note:* Outcome definition: Global Aesthetic Improvement Scale score ≤ 2 (“much improved” or “very much improved”). Reference groups: Age ≥ 40 years; NRS score 4–10 (moderate to severe pain).

Monopolar RF is effective for skin tightening and wrinkle improvement. In younger individuals, dynamic wrinkles are more common, accompanied by milder skin laxity, making them more responsive to noninvasive treatments. In contrast, older patients with severe laxity and deep wrinkles may benefit more from surgical interventions. These findings align with the consensus that ideal candidates for monopolar RF treatment should have no more than mild to moderate skin laxity [4]. Additionally, discomfort and pain during treatment do not correlate with better outcomes. Pain perception depends on various factors, including pain tolerance, energy level, and treatment location. It is important to monitor patients' feedback and adjust energy settings accordingly, as increased pain may raise the risk of adverse events. The results were observed in both groups, indicating that it was universal across monopolar RF devices rather than a specific device.

However, this study has several limitations. Firstly, this is a post hoc analysis; therefore, the results are exploratory rather than confirmatory. Secondly, the small proportion of male subjects limits the ability to analyze gender disparities. Thirdly, several important confounders were not fully examined, including baseline skin laxity severity, treatment energy parameters, facial region, and skin thickness. Additionally, the difference observed at 30 days posttreatment was not statistically significant and was therefore excluded from the table. This is likely because the efficacy of monopolar RF had not yet been fully established at the 30‐day time point, which aligns with the known time course of neocollagenesis following RF treatment. Further larger, prospective studies designed to address these factors are needed.

In conclusion, our analysis demonstrates that younger participants are more likely to benefit from RF treatment, and pain perception during treatment is not associated with its efficacy. These findings highlight the importance of considering age when managing patient expectations prior to treatment. Furthermore, more severe pain should not be interpreted as an indicator of greater efficacy; therefore, alleviating pain and improving comfort during treatment may not affect the outcome.

## Funding

The authors have nothing to report.

## Ethics Statement

All procedures performed in studies involving human participants were in accordance with the ethical standards of the institutional and/or national research committee and with the 1964 Helsinki Declaration and its later amendments or comparable ethical standards. All procedures were approved by the Ethics Committee.

## Consent

Informed consent for procedure, photos, and publication was obtained from all subjects involved in the study.

## Conflicts of Interest

The authors declare no conflicts of interest.

## Data Availability

The data that support the findings of this study are available on request from the corresponding author. The data are not publicly available due to privacy or ethical restrictions.

## References

[1] T. W. Griffiths , R. E. B. Watson , and A. K. Langton , “Skin Ageing and Topical Rejuvenation Strategies,” British Journal of Dermatology 189, no. Suppl 1 (2023): i17–i23, 10.1093/bjd/ljad282. 37903073

[2] G. K. Austin , S. L. Struble , and V. C. Quatela , “Evaluating the Effectiveness and Safety of Radiofrequency for Face and Neck Rejuvenation: A Systematic Review,” Lasers in Surgery and Medicine 54, no. 1 (2022): 27–45, 10.1002/lsm.23506. 34923652

[3] Z. Wang , T. Li , Q. Wa , Z. Liu , B. Yang , and Y. Yan , “Efficacy and Safety of a Novel Monopolar Radiofrequency Device for Skin Tightening: A Multicenter, Randomized, 6‐Month, Assessor Blind, Positive Parallel‐Controlled, Non‐Inferiority Trial,” Lasers in Medical Science 41, no. 1 (2026): 39, 10.1007/s10103-026-04841-4. 41758257 PMC12948897

[4] A. Chapas , B. S. Biesman , H. H. L. Chan , et al., “Consensus Recommendations for 4th Generation Non‐Microneedling Monopolar Radiofrequency for Skin Tightening: A Delphi Consensus Panel,” Journal of Drugs in Dermatology 19, no. 1 (2020): 20–26, 10.36849/jdd.2020.4807. 31985194

